# Socioeconomic influences on survival outcome in idh-wildtype glioma patients: examining the role of age, education, and lifestyle factors

**DOI:** 10.1007/s00701-025-06594-5

**Published:** 2025-06-27

**Authors:** Matthias Demetz, Aleksandrs Krigers, Julia Klingenschmid, Claudius Thomé, Christian F. Freyschlag, Johannes Kerschbaumer

**Affiliations:** Department of Neurosurgery, Medical University of Innsbruck, Innsbruck, Austria

**Keywords:** Socioeconomic factors, Glioblastoma, Lifestyle factors, Education, IDH wildtype

## Abstract

**Background:**

Socioeconomic factors influence survival in different cancer types. This study aimed to assess the impact of various socioeconomic factors, including education, marital status, lifestyle, and social network, on survival in patients with Isocitrate dehydrogenase (IDH) wildtype gliomas.

**Methods:**

We conducted a retrospective analysis of 309 adult patients with de novo diagnosed IDH wildtype gliomas who underwent surgical treatment at our institution between 2014 and 2022. Socioeconomic factors, including education, marital status, employment, language, lifestyle (alcohol abuse, smoking, BMI), and social network were assessed. Progression-free survival (PFS) and overall survival (OS) were analyzed using Kaplan–Meier and Cox regression models.

**Results:**

Education, alcohol abuse, and age significantly influenced OS. Patients with higher education had significantly lower tumor volumes (*p* = 0.037) and longer survival (*p* = 0.001). Alcohol abuse was associated with significantly shorter OS (8.6 vs. 17.9 months, *p* = 0.034). Patients aged over 65 years showed significantly shorter OS in this study (14.9 months vs 33.2 months, *p* < 0.001). Native and non-native German speakers had similar outcomes. Marital status did not significantly affect survival.

**Conclusion:**

Socioeconomic and lifestyle factors, especially higher education and alcohol abuse, significantly impact survival in IDH wildtype glioma patients. These findings suggest that, in addition to molecular features and oncological treatment, socioeconomic and lifestyle factors also play a crucial role in influencing the prognosis of patients with IDH wildtype gliomas.

## Introduction

Advances in the molecular understanding of glioblastoma multiforme (GBM) over the past decades have provided significant insights that are reshaping prognostic predictions for this tumor[8, 13, 14, 44]. These molecular insights are complemented by advancements in surgical techniques. Studies have shown that greater extent of resection (EOR) is correlated with improved survival and delayed recurrence, underscoring the importance of surgical advancements and precision in GBM management[5, 9, 11]. Despite these advances, however, survival remains limited, and the role of different socioeconomic factors in mediating access to and engagement with these therapies is still unclear, suggesting a need for a more comprehensive understanding of non-medical determinants in GBM outcomes[19, 35, 41].

In other malignancies, studies have consistently highlighted that socioeconomic determinants such as education, lifestyle behaviors, and social support networks can have significant impacts on survival outcomes[17, 29, 45]. Patients with lower education levels may have limited access to healthcare information, which can reduce their likelihood of engaging with adjuvant therapies[32, 33]. Similarly, alcohol abuse and inadequate social support networks are known to negatively impact patients’ willingness to pursue or adhere to treatments, subsequently affecting their survival[18, 38, 42]. These factors contribute not only to disparities in access to treatment but also to variations in treatment compliance and overall health maintenance[42]. These findings suggest that socioeconomic determinants may play a critical role in shaping patients’ choices and, ultimately, their outcomes. Furthermore, socioeconomic factors may also influence clinical decision-making, potentially affecting treatment choices, access to specialized care, and patient adherence to therapeutic recommendations.

This study aimed to examine the impact of specific socioeconomic factors, including age, education, marital and employment status and substance use, on progression-free survival (PFS) and overall survival (OS) in glioblastoma patients. By elucidating the relationship between these socioeconomic variables and survival outcomes, we hope to provide insights that may inform not only clinical treatment strategies but also supportive care initiatives tailored to patients facing socioeconomic barriers.

## Materials and methods

In this study, all adult patients (aged ≥ 18 years at the time of surgery) who underwent their first surgical procedure (either resection or biopsy) for a IDH wildtype intracranial glioma, between June 2014 and December 2022 at the authors’ institution were analyzed retrospectively.

Surgical resection was performed as the standard of care for all eligible patients at our institution. For patients with gliomas in non-resectable locations, biopsy followed by radio-chemotherapy was conducted[40]. Postoperative follow-up was scheduled at three-month intervals. Tumor progression was assessed according to the Response Assessment in Neuro-Oncology (RANO) criteria[3]. Patients'general condition was evaluated pre- and postoperatively using the Karnofsky performance status (KPS) and the Clinical Frailty Scale (CFS). Tumor volumes were manually segmented using ITK-SNAP software (v.3.8.0 for Mac OS, UPenn and UNC dev.) in various MRI sequences.

Neuropathological assessment was routinely conducted on formalin-fixed paraffin (FFP)-embedded tissue by a team of experienced neuropathologists. Histological diagnoses were made according to the 2021 revised 6th edition of the WHO grading system for central nervous system tumors[28]. For patients initially diagnosed according to the 2016 WHO classification, diagnoses were updated to align with the criteria of the 2021 WHO classification. IDH1 status at the R132H position was tested using immunohistochemistry (IHC), and for patients under 40 years of age with a negative result, DNA sequencing was performed to confirm IDH1 and IDH2 wildtype status. Nuclear alpha thalassemia/mental retardation X-linked (ATRX), epidermal growth factor receptor (EGFR), and MIB-1 expression as a proliferation marker were also evaluated using IHC. For cases where IDH, ATRX, or EGFR had not been initially assessed and sufficient tissue was available, these markers were re-evaluated.

To assess patients'social networks, educational background, and socioeconomic status, we retrospectively reviewed data at the time of surgical intervention on marital status, native language, immigration background, employment status, and health insurance type (private vs. public). Immigration background was assessed together with native language and other social determinants as part of the routine preoperative anamnesis conducted during the clinical admission. Social networks were also evaluated during routine pre- and postoperative anamnesis as part of the clinical planning process. This included discussions with the patient regarding family members, partners, or close contacts who could be involved in care and support during treatment and hospitalization. This information was used to help coordinate the patient's admission and ensure adequate social support. Educational attainment was categorized into three groups based on each patient’s highest level of education: university/college degree, completed high school, and did not complete high school. Lifestyle factors were evaluated by analyzing patients'histories of alcohol abuse, smoking status, and body mass index (BMI). Alcohol use, along with smoking habits, was routinely assessed during the standard preoperative admission process for all patients. In addition, we reviewed patients’ medical records for any documented history of alcohol abuse and screened for typical alcohol-related comorbidities, such as liver cirrhosis, as noted by other departments.

Austria's health insurance system is universal and mandatory, funded through income-based contributions, and provides comprehensive coverage for medical services; private health insurance is available for additional benefits, such as more private hospital rooms.

Data analysis was conducted using IBM SPSS Statistics (Version 27.0 for Mac OS; Armonk, NY: IBM Corp.). Scale variables were evaluated with T-tests and presented as mean ± standard deviation (SD) when normally distributed, or with the Mann–Whitney U-test, shown as median with interquartile range (IQR), when normal distribution was not met. Categorical variables were compared using the Chi-squared test. Progression-free survival (PFS) and overall survival (OS) were estimated with Kaplan–Meier analysis and compared using the Log-Rank test. Cox regression analysis was performed to calculate hazard ratios (HR) for oncological progression or death. The significance level (α) was set at 0.05, and 95% confidence intervals were reported.

This study was approved by the Ethics Committee of the Medical University of Innsbruck (Approval Number: 1291/2024) and was conducted in accordance with the ethical standards outlined in the 1964 Declaration of Helsinki and its subsequent amendments.

## Results

### Patient characteristics

313 patients (176 male, 137 female) with a median age of 66 years (IQR 58–74, absolute range 20–88) at the time of surgery met the inclusion criteria.

Baseline socio-economic characteristics can be found in Table 1.

**Table 1:** Socioeconomic and lifestyle data including marital status, employment status, highest accomplished education, alcohol and nicotine consumption

	Yes	No
Married at time of surgery	76.7%	23.3%
Native German speakers	89.5%	10.5%
Unemployed at time of surgery	1.3%	98.7%
Private health insurance	22%	78%
High school degree	58%	42%
University/college degree	13.8%	86.2%
History of alcohol abuse	3.5%	96.5%
Active smokers at time of surgery	16.3%	83.7%
Preoperative epileptic seizures	23.6%	76.4%

Married patients were significantly more likely to have a high school degree (*p* = 0.024). Patients who were covered by private health insurance were more likely to have a high school degree (*p* = 0.006) or a university degree (*p* = 0.001). Patients who completed high school were significantly less likely to be unemployed (*p* = 0.018). We could not find any significant differences between patients with higher and lower education in terms of adjuvant treatment (*p* > 0.05). Male patients were significantly more like to suffer from alcohol abuse (*p* = 0.025). However, in our study, patients with alcohol abuse did not show a significant difference in the rate of receiving adjuvant therapy (*p* > 0.05). The median BMI of the cohort was 25.5 (IQR 22.6–28.4). The median preoperative KPS was 90 (IQR 80–100), the median preoperative CFS was 3 (IQR 2–4).

### Imaging and treatment

A total of 248 patients (79.2%) underwent surgical resection, while in 65 patients (20.8%) only biopsy was performed due to tumor location. In 161 patients (51.8%), the glioma was located in an eloquent area of the brain, and 35 patients (11.2%) had a multifocal tumor at the time of diagnosis. Tumor laterality was balanced, with 50.5% of gliomas located in the right hemisphere and 49.5% in the left hemisphere. The most common tumor location was the temporal lobe, affected in 124 patients, followed by the frontal lobe, which was involved in 123 cases. The median preoperative tumor volume on T1 contrast-enhanced (CE) MRI was 22.9 cm^3^, while the median volume on FLAIR-weighted MRI was 49.2 cm^3^. Patients with higher education levels had significantly lower preoperative tumor volumes (*p* = 0.037). Complete resection of the contrast-enhanced tumor was achieved in 158 patients (51.1%), while supratotal tumor resection (complete FLAIRectomy) was performed in 29 patients (9.4%).

### Histopathological and molecular characteristics

In our cohort, 302 patients (96.5%) had an initial diagnosis of WHO Grade 4 glioma. 11 patients (3.5%) were initially diagnosed with WHO Grade 3 gliomas, which were subsequently corrected to WHO Grade 4 based on the 2021 classification. EGFR overexpression was observed in 53% of the cases. ATRX expression was present in 293 patients (93.6%), lost in 9 patients (2.9%), and could not be assessed in 11 cases (3.5%). The median MIB-1 index was 30% (IQR 19–41).

### Survival and outcomes

The median postoperative KPS amounted to 90 (IQR 80–100), the median postoperative CFS stayed at 3 (IQR 2–4). A total of 234 patients (75.7%) received adjuvant therapy, while 75 patients (24.3%) did not, either due to low performance status or based on the patient's wishes. Tumor treating fields (TTF) were used by 34.5% of our patients. At the first follow-up, the results for KPS and CFS remained unchanged from postoperative values.

The mean PFS for our cohort was 13.4 months (SD 0.9). In the Kaplan–Meier analysis, PFS was significantly influenced by higher age, with older patients showing a shorter time to progression. However, none of the socioeconomic characteristics or lifestyle factors assessed, including marital status, educational background, alcohol abuse, smoking status, or body mass index, had a significant impact on PFS.

**Fig. 1:**
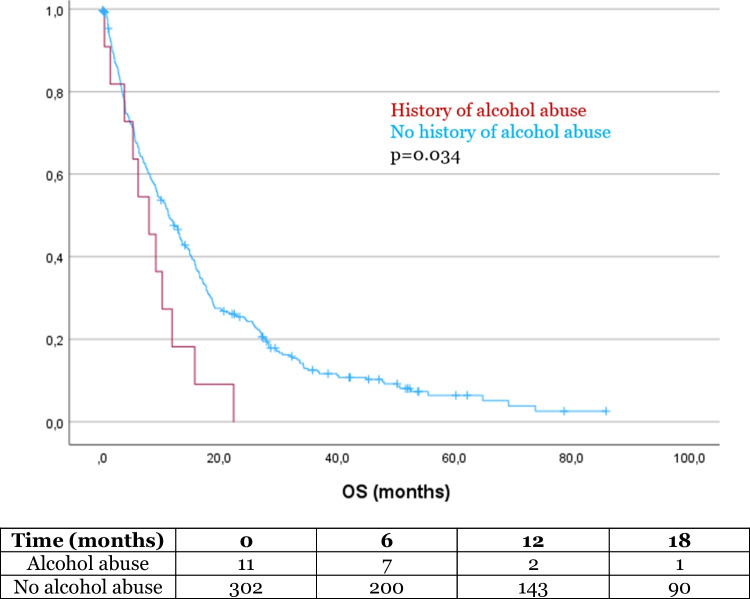
Patients with history of alcohol abuse showed significantly worse outcome

**Fig. 2:**
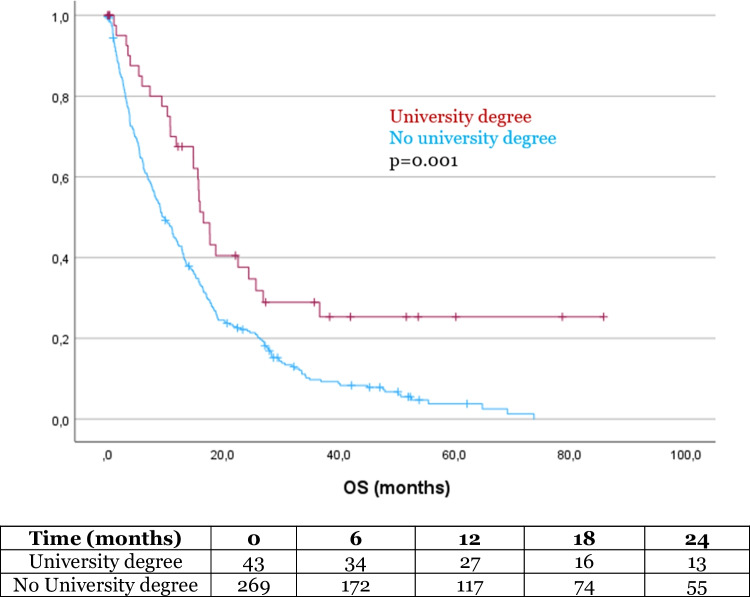
Patients with high education status (university degree) showed significantly more favorable OS

**Fig. 3:**
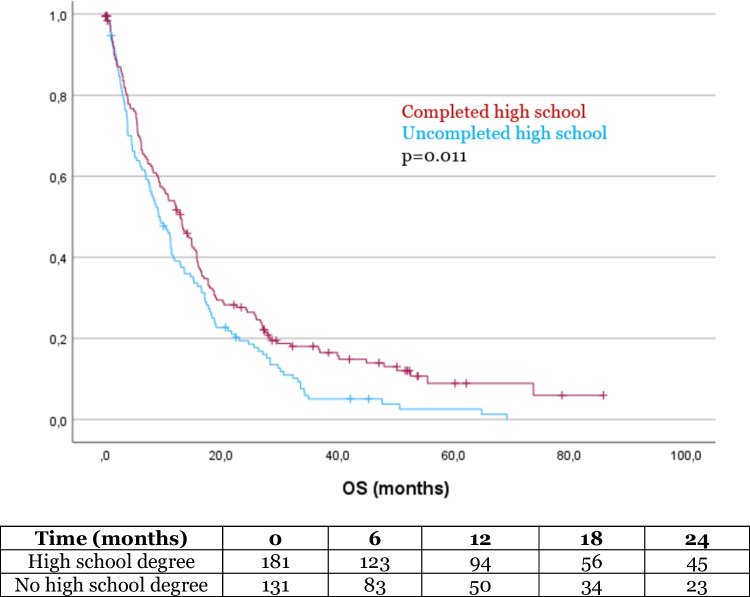
Patients with completed high school showed significantly better outcome compared to patients with no high school degree

**Fig. 4:**
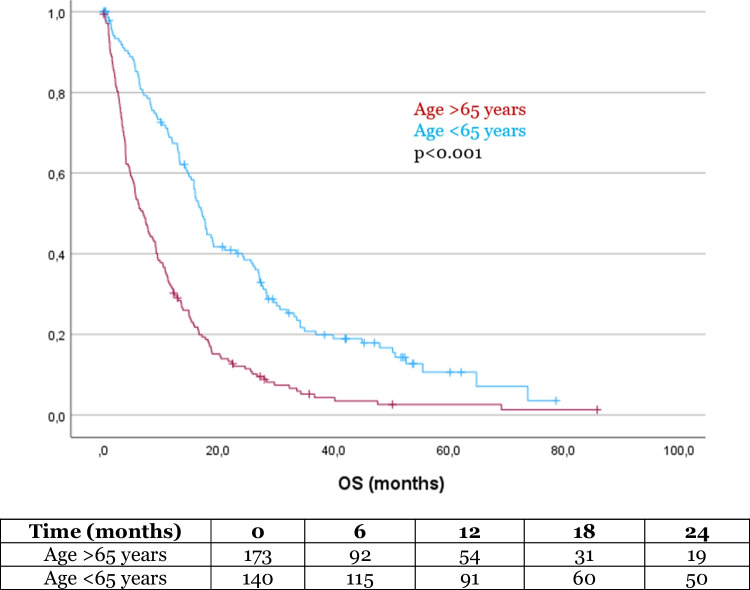
Patients aged 65 years or older showed significantly worse outcome

The mean OS in our cohort was 17.5 months (SD 1.2). In the Kaplan–Meier analysis, OS was significantly influenced by several factors. Patients with a history of alcohol abuse had a significantly shorter survival compared to those without alcohol abuse (8.6 months vs 17.9 months, *p* = 0.034, Fig. 1). Additionally, patients with a university degree had a significantly longer OS compared to those without a degree (32.5 months vs 15.5 months, *p* = 0.001, Fig. 2). Similarly, patients who completed high school had a longer OS than those who did not (20.5 months vs 13.9 months, *p* = 0.011, Fig. 3). Age also had a significant impact on survival, with patients over 65 years of age experiencing worse outcome compared to younger patients (14.9 months vs 33.2 months, *p* < 0.001, Fig. 4). However, no significant influence on OS showed marital status, immigration background, smoking, BMI, employment status and health insurance type. The statistically significant results for alcohol abuse, educational level, and age remained consistent in the univariate analysis (*p* < 0.05).

In multivariate analysis, both lower preoperative tumor volume (*p* = 0.013) and a higher educational degree (*p* = 0.001) remained significant factors for improved overall survival.

All significant differences in overall survival related to age, alcohol abuse, and educational level remained statistically significant after adjustment for MGMT promoter methylation status and EOR.

## Discussion

Socioeconomic factors, including social networks and lifestyle, are increasingly recognized as critical influences on survival in cancer patients[17, 29, 45]. A robust social network can play a pivotal role in supporting patients throughout treatment, particularly in helping them withstand and actively participate in adjuvant therapy. This support may take many forms, such as practical assistance, emotional encouragement, and even increased adherence to medical advice, all of which can contribute positively to treatment outcome[1, 4, 7, 24, 37]. In other malignancies, factors like higher education, stable employment, and a supportive community have been linked to improved survival and greater willingness to pursue intensive therapies[12, 21, 31]. However, in glioma patients, the impact of socioeconomic factors remains less well-defined[4, 19]. Unlike many other cancers, gliomas often present unique neurological and cognitive challenges, which can complicate both the assessment of social support and the patient’s capacity to participate in therapy[20]. The aim of this study was therefore to assess the impact of various socioeconomic factors on survival in patients with IDH wildtype gliomas.

In this study, we observed significant influences of socioeconomic factors on survival in patients with IDH wildtype gliomas. While PFS was notably impacted by age, socioeconomic factors did not show a direct effect on time to progression. However, OS was significantly affected by a range of socioeconomic and lifestyle characteristics: patients with a history of alcohol abuse had a significantly shorter OS. In contrast, patients with higher educational attainment, including both high school and university degrees, demonstrated significantly longer OS. Age was also a significant predictor, with older patients experiencing shorter OS.

Lifestyle factors demonstrated varying impacts on survival in our cohort. Alcohol abuse was associated with significantly reduced OS, which may be attributed to a weaker social network, reduced access to support, and potentially limited compliance with treatment due to alcohol dependency[30]. Alcohol abuse was a significant factor not only in univariate, but also in multivariate analyses and should therefore be considered a relevant prognostic factor in patients with glioma. This finding aligns with observations in other cancer populations, where alcohol abuse can negatively impact both physical and psychosocial resilience[26, 38]. In contrast, other lifestyle factors, such as smoking status and higher BMI, did not show any significant influence on survival in our patients. These results suggest that, unlike alcohol abuse, factors such as smoking and higher BMI do not appear to impact patients'social networks or compliance with treatment and therefore do not significantly affect survival outcomes in glioma patients. However, we found no significant difference regarding adjuvant therapy including TTF between patients with alcohol abuse and without alcohol abuse, indicating that the shorter OS observed in these patients cannot be fully attributed to differences in treatment rates. This suggests that other underlying factors related to alcohol abuse may contribute to reduced survival, warranting further research to better understand these complex interactions in glioma patients. Our data however suggest that patients with a history of alcohol abuse should be followed up more closely due to their significantly worse overall survival. Closer monitoring and tailored interventions could help mitigate these factors and improve outcomes in this vulnerable patient group.

Patients with higher education levels showed significantly lower preoperative tumor volumes, which may indicate that they tend to seek medical consultation earlier, potentially due to better disease awareness and health literacy[27, 36]. This earlier detection could result in a more favorable clinical presentation at the time of surgery and favor complete resection. Despite these advantages, we found no significant differences in the receipt of adjuvant treatment between patients with varying education levels, suggesting that factors beyond early detection and social support may influence treatment decisions.

In our study, marital status did not significantly influence patient outcome, suggesting that the presence of a spouse or partner alone may not be a key determinant of survival. While it is well-established that strong social networks are important for supporting patients through treatment, our findings indicate that non-married patients who have established partnerships or close relationships with family and friends also demonstrate favorable outcome[10, 22]. Therefore, a supportive environment, whether through a spouse, family, or friends, can provide the emotional and practical resources needed to navigate the challenges of glioma treatment, ultimately contributing to better outcome.

Higher age was a significant factor influencing survival in our study, with older patients experiencing poorer outcome. This is consistent with previous findings in glioma patients[6, 16, 23, 34]. In developed countries, where life expectancy is increasing, the incidence of gliomas in older adults is expected to rise, making it increasingly important to understand the impact of age on treatment outcome and survival[23]. Older patients may face additional challenges, such as comorbidities, frailty, and reduced treatment tolerance, which can complicate their ability to undergo aggressive therapies and affect their overall prognosis[25, 43]. As the demographic shift towards an older population continues, these insights will be crucial for tailoring treatment strategies and providing appropriate care to this growing cohort of glioma patients.

In our study, we did not find any significant differences in survival between native German speakers and non-native German speakers with an immigration background. While language barriers may pose challenges in communicating complex medical information, the techniques employed in our institution, such as the use of professional translation services and the active involvement of family members in discussions, seem to mitigate these difficulties effectively[2, 15, 39]. These strategies appear to ensure that non-native speakers receive the necessary information to make informed decisions regarding their treatment, allowing them to engage in therapy and adhere to medical recommendations in a similar manner to native speakers. This suggests that with the right support systems in place, language differences do not necessarily impede the quality of care or impact outcome in glioma patients.

This study has limitations that should be considered. First, the retrospective study design should be mentioned. Additionally, while we assessed various socioeconomic factors such as education, marital status, and employment, due to the retrospective design we were unable to evaluate the financial status and income of the patients, which has been an important variable in previous studies on cancer outcomes. Finally, our findings are based on a single-center cohort, and prospective multicenter studies are needed to validate these results and provide a broader understanding of the impact of socioeconomic factors on glioma outcomes.

## Conclusion

In conclusion, our study highlights the significant impact of socioeconomic and lifestyle factors, such as higher education, alcohol abuse and age, on survival in patients with glioblastoma. While some factors, like education, may influence timely diagnosis and treatment adherence, others such as alcohol abuse, underscore the importance of social networks and compliance in patient outcomes. These findings suggest that addressing these factors in clinical practice could improve outcome, and further multicenter, prospective studies are needed to confirm these results and explore the underlying mechanisms in greater depth.

## Data Availability

No datasets were generated or analysed during the current study.
